# Lived experiences for individuals with cystic fibrosis who have undergone lung transplantation: a qualitative study

**DOI:** 10.1186/s12912-025-02774-x

**Published:** 2025-02-03

**Authors:** Ulrika Skogeland, Isabelle de Monestrol, Terezia Pincikova, Tove Godskesen

**Affiliations:** 1 Department of Pediatrics, Cystic Fibrosis Centre, CLINTEC, Karolinska Institutet, Karolinska University Hospital, Stockholm, Hälsovägen, Stockholm, 141 86 Sweden; 2 Faculty of Nursing and Health Sciences, Nord University, Box 1490, Bodø, 8049 Norway; 3 Centre for Research Ethics & Bioethics, Department of Public Health and Caring Sciences, Uppsala University, Box 564, Uppsala, 751 22 Sweden

**Keywords:** Cystic fibrosis, Lung transplantation, Qualitative research, Individual interviews, Nurses, Nursing

## Abstract

**Background:**

Cystic Fibrosis (CF) significantly affects the respiratory system, often requiring lung transplantation in advanced stages. This life-saving procedure presents substantial challenges and uncertainties. While there is existing research on support and information needs post-lung transplant from various perspectives, this study aims to specifically address the unique experiences and challenges faced by individuals with CF during both the pre-transplant and post-transplant periods.

**Methods:**

Twenty-three lung-transplanted individuals with CF participated in this exploratory qualitative study. Data was collected through individual semi-structured interviews and analyzed using inductive content analysis.

**Results:**

Participants faced physical and mental challenges, including fatigue, depression, and anxiety. The waiting period involved isolation, dependence on family, and guilt. Post-transplant, they dealt with relief but also severe pain and adjusted to a new identity. Participants highlighted the importance of taking immunosuppressive medications as prescribed, even though the regimen was complicated and these medications had side effects. Participants stressed the need for earlier and more open dialogue with healthcare professionals and better emotional preparation for the transplant process, including preparedness for pain and previously inadequately addressed concerns such as depression and anxiety.

**Conclusions:**

This study underscores the significant physical and emotional challenges individuals with CF face during lung transplantation, highlighting the need for comprehensive, person-centered care. Psychological support, effective post-transplant pain management, and early palliative care may be beneficial approaches to improve the patient experience. Nurses can play a pivotal role in this process by ensuring clear communication, managing pain, educating patients on immunosuppressive regimens, and advocating for holistic care.

**Supplementary Information:**

The online version contains supplementary material available at 10.1186/s12912-025-02774-x.

## Background

Cystic Fibrosis (CF) is an autosomal recessive disease. In Sweden, it affects 1 of every 5600 births and is thus the most common life-shortening genetic disorder [1]. The genetic mutation affects the CF transmembrane conductance regulator (CFTR), which regulates anion transport and mucociliary airway clearance [2, 3]. This dysfunction leads to thick mucus in the respiratory and gastrointestinal system, causing a broad spectrum of symptoms and infections [4].

Medical advancements—such as infection control, nutritional support, centralized CF care, and improved physiotherapy—have gradually extended the lives of individuals with CF. The average lifespan of individuals with CF was up to recently 45 to 50 years [5], with respiratory insufficiency remaining the leading cause of mortality [6].

CFTR modulators represent new therapies targeting the basal defect of CF, including the triple-combination drug Elexacaftor/Tezacaftor/Ivacaftor (known as Trikafta^®^ in the USA and Kaftrio^®^ in Europe). They have improved lung function and reduced hospitalizations in responsive individuals [7, 8]. These therapies have brought new hope for a better prognosis of CF [6], and the median predicted survival age has increased to 61.4 years for individuals born between 2019 and 2023 in the United States [9]. However, not all individuals can benefit from these therapies. Up to 10–15% of individuals with CF worldwide have genetic variations that make the drugs ineffective. Some experience side effects like liver damage and cannot continue with the CFTR modulator therapy. Thus, while these therapies offer hope for some, they are not suitable for everyone [10].

For individuals with advanced CF and respiratory insufficiency, lung transplantation is often the only life-saving treatment. This procedure does not only significantly improve survival rates but also enhances quality of life by alleviating respiratory symptoms and promoting overall health [3, 11]. However, lung-transplanted individuals must take lifelong immunosuppressive medications, and failing to adhere to this medical regimen can cause severe complications, including transplant failure and rejection [12, 13]. The immunosuppressive medications cause side effects varying from mild to severe. These include discomfort, pain, changes in hair growth or hair loss, weight gain, skin issues such as acne, tremors, nausea, sleep problems, increased anxiety, and depression. The medications suppress the immune system, which can result in more severe side effects, including increased susceptibility to infections and a higher risk of cancer [14, 15]. Additionally, due to the pharmacology of immunosuppressive medications, kidney insufficiency may occur, sometimes requiring kidney transplantation [11, 16].

A previous study highlights the emotional complexity and challenges that individuals with CF face when they undergo lung transplantation [17]. These challenges, both before and after the procedure, include stress, guilt, apprehension, and a sense of worthlessness.

While significant research has been conducted on CF and lung transplantation, the psychosocial and emotional challenges specific to individuals with CF remain underexplored. Existing literature on general lung transplant populations highlights a range of significant psychosocial concerns, such as anxiety, depression, and social reintegration, which are also pertinent to those with CF [11, 17–20]. Notably, some of these include participants with CF, yet they do not always provide detailed insights [21]. Thus, this study aims to explore the lived experiences of individuals with CF who have undergone lung transplantation.

## Methods

### Design

This study employed an exploratory qualitative design and is reported according to COREQ’s guidelines [22].

### Study setting and recruitment

CF centers are located within four university hospitals in Sweden. Two university hospitals perform all lung transplantations in Sweden. CF centers care for lung transplanted individuals and gradually assume responsibility for post-transplantation care and check-ups.

Lung transplanted individuals with CF aged 18 years or older who could fluently understand Swedish or English were included in this study. Those deemed too sick to complete interviews were excluded. Participants were recruited from Sweden’s largest and smallest CF centers. US and another registered nurse contacted the presumptive participant during clinic appointments or via phone calls when no scheduled appointments were imminent. All were verbally informed about the study’s purpose and procedures, and voluntary participation was emphasized. Written information about the research was provided, along with the opportunity to ask questions and a consideration period of two days or more, if required. Before the study started and before giving written consent, they were informed again that participation was voluntary and that they could withdraw without explanation and with no negative consequences. They could choose the interview time and location to ensure a comfortable and accommodating environment. All participants were interviewed alone to create a comfortable environment where participants felt free to share openly.

### Participants

Twenty-seven individuals were asked to participate in the study: four declined, two from each center. Subsequently, twenty-three participants consented to participate, comprising 12 females and 11 males, with an average age of 43 years (± 19). The participants were, on average, 11 years post-lung transplantation, with individual durations ranging from 10 months to 19.25 years. The average waiting time was eight months, spanning a minimum of 8 days to a maximum of 30 months. At the time of transplantation, the average age of participants was 34 years, ranging from 17 to 55.

### Data collection

US collected data. A semi-structured interview guide (Table 1) with open-ended questions allowed participants to share their experiences and perspectives. Interviews were conducted between October 2022 and April 2023, either in a quiet room at the hospital (*n* = 14) or through the digital Teams platform (*n* = 9), according to the participants’ preferences and because many participants resided a considerable distance from the CF center. After interviewing the final five participants, data saturation was reached. This means that the collected data became repetitive and did not contribute anything new to the results. The interviews ranged from 12 to 46 min, and professional transcriptionists audio-recorded and transcribed them verbatim.

**Table 1:** Interview guide

• How long did you wait for lung transplantation?
• Can you describe how you decided to undergo transplantation?
• Could you share your overall experiences during the waiting period?
• What were the most challenging aspects for you?
• How do you perceive your family’s or relative’s experiences during this time?
• What kind of information were you provided with while waiting?
• What support did you need while waiting for a lung transplantation?
• Did you perceive any aspects that health care professionals may not have fully considered in your care, such as cultural, religious, or life philosophy factors?
• How do you feel about taking immunosuppressive medications daily?
• Is there anything else you want to discuss that we have not discussed?

### Data analysis

Data analysis followed the inductive content analysis approach [23]. Initially, US listened to all interviews multiple times to gain a general understanding of the participants’ experiences. The interview transcripts were read numerous times to identify meaning units comprehensively. Short notes (codes) were generated during the coding process, and relevant statements or concepts related to the study’s purpose were underlined in the text using Excel and Word. Similarities, differences, and patterns between the categories were examined, and similar subcategories were initially merged to create a more concise set of categories, as shown in Table 2. TG read eleven interviews to ensure quality and consistency. Discussions between US and TG helped develop the analysis and reach a consensus on the final categories. Similar subcategories were merged during these discussions, and irrelevant or redundant categories were removed. After finalizing the categories, all authors independently reviewed the analysis and engaged in discussions until a consensus was reached on the final version.

**Table 2:** A representative example of the interview analysis process

Meaning unit	Condensed meaning unit	Code	Subcategory	Category
I was focused on being transplanted. My goal was to be transplanted and given a second chance. I trusted the surgeons, and they were allowed to do what they wanted if I was transplanted with new lungs.	I would be transplanted and given a second chance for a new life	Puts trust in the hands of surgeons for a second chance	A second chance but with new challenges	Reborn with new lungs

### Ethical considerations

Confidentiality was ensured by removing all personal identifiers from the transcribed interviews and replacing them with pseudo-anonymized codes. Participants were referred to as “P,” followed by a unique code number ranging from 1 to 23. This precautionary measure ensured that interviewees could not be identified. The code key was securely stored on a password-protected computer and kept separate from the interview data. Only the first author had access to the code key. The coded data was used during the analysis, and the findings were reported in a way that made it impossible to identify the participants involved.

### Rigor and reflexibility

US and TG developed the interview guide. The guide was reviewed by healthcare professionals, including two RNs and a psychologist with clinical experience in CF care. Two individuals with CF who had undergone transplantation, one newly transplanted and one transplanted some years ago, provided valuable input to the interview guide. This review process expanded the questions concerning side effects and the support given to relatives, thus contributing to the overall rigor of the study. Before the interviews, two comprehensive pilot interviews were conducted with individuals who had undergone lung transplantation to ensure the interview guide was appropriate and the study was well-prepared. No further changes were made. The research team consisted of two RNs with extensive experience in CF care. Two authors were MDs specializing in respiratory diseases and engaged in reflexivity during the analysis to address potential biases.

## Results

Three main categories were identified: (1) Finally, on the waiting list, (2) Reborn with new lungs, and (3) A new life with medications and their effects. These categories were further divided into seven subcategories (Table 3).

**Table 3:** An overview of categories and subcategories in the qualitative analysis

Categories	Subcategories
Finally, on the waiting list	• *Transitioning and coping with deterioration* • *Dependency*,* isolation and guilt* • *The role of communication*
Reborn with new lungs	• *A second chance with new challenges* • *Adjusting to a new identity*
A new life with medications and their effects	• *Emphasizing the importance of medications* • *Burden with side effects*

### Finally, on the waiting list

#### Transition and coping with deterioration

Most participants described being unprepared for the decline in their lung function, which eventually led them to need a lung transplant. The transition to a deteriorated state varied among participants. For instance, one participant noted:

“I developed a pneumothorax and became very ill, with my condition worsening rapidly… I was shocked to find that I couldn’t walk up three steps or make my bed. My health deteriorated quickly, and I was confined to my apartment. During the last few weeks in the hospital, I was barely aware of my surroundings.” (P18).

For others, the decline was gradual, spanning several years:

“It developed over three to four years, and the first real memory was meeting Dr. X, who told me bluntly when she saw my spirometry: ‘How does it feel to only have 33% lung capacity?’ I thought I had around 53% because I added FEV and FVC and came to about 50. However, she said it is the FEV they follow, and I was approaching the 30% mark. That left an impression on me for a while, but I still felt fine…” (P9).

When participants reached a level of health deterioration to the point of needing a transplant, many felt unprepared, believing that transplantation was a distant possibility, if necessary at all. However, the transplantation and CF teams conducted rigorous check-ups before transplantation to cover all medical aspects, making them feel safe and well-cared for.

“I had for many years thought that I would die from CF and that there would not be a transplantation, that I would die around forty.” (P16).

As the participants’ condition worsened, they experienced a substantial decrease in strength and mobility. For some, this decline resulted in overwhelming fatigue, which significantly impacted their daily activities.

“I was so ill then, you know, just taking a shower was like a marathon. So, I mostly stayed home. I tried shopping but was completely exhausted afterward.” (P20).

Participants also struggled with their daily routines:

“I would work on Monday, rest on Tuesday and Wednesday to build up the strength for work on Thursday, and then rest on Friday, Saturday, and Sunday to get through the following week.” (P23).

The constant physical discomfort and the restrictions imposed by their symptoms took a significant toll on their mental health, resulting in depression, anxiety, or feelings of hopelessness. As the severity of symptoms escalated, participants described an increasing sense of helplessness and of feeling overwhelmed by their inability to control their bodies. This sense of loss of control led to heightened anxiety, with participants expressing concern over the progression of their condition and the uncertainty surrounding their future.

“I stayed awake at night because even a slight cough would lead to bleeding. It is not something I think about now, but at that time, it was a tough, tense, and depressing period.” (P23).

The decline caused by CF, with its demanding symptoms, made this period of uncertainty particularly challenging.

“The battle was against the clock. Will I drown in my mucus before I get new lungs.” (P23).

Cognitive impairment posed a considerable challenge, with participants often struggling to understand the information provided by healthcare professionals fully. The constant feelings of exhaustion and breathlessness led to a significant amount of stress:

” At that time, it was like… I had to walk from the station to the hospital, and I needed to have done my inhalation properly for a week before so I could manage it and at the same time be mentally alert enough to undergo these examinations and speak for myself.” (P16).

Maintaining hope was crucial during these challenging times. The waiting period felt like an emotional rollercoaster of hope and despair. Participants frequently longed to perform everyday tasks without the constraints imposed by their compromised respiratory health. Simple activities, such as doing the laundry, held significant meaning for them.

“My life was about taking medication, inhalation, and breathing exercises. My motivation was to breathe without oxygen. I wanted a normal life that gave me strength and motivated me to struggle.” (P17).

#### Dependency, isolation, and guilt

At times, participants isolated themselves to avoid infections. This isolation meant they hardly met anyone, which was incredibly challenging for teenagers living with their families, as they wanted to do what their friends did. Participants expressed their dependence on various tasks, such as household chores and transportation to medical appointments.

“Our sons often had trouble socializing with friends and having them over at our house. We did not realize this could be so challenging.” (P12).

Participants often felt isolated and emotionally burdened due to the need to avoid infections, which limited their social interactions and increased their dependency. Consequently, they depended on family members for physical, emotional, or medical support. Some participants recognized that the dependency fostered deep connections and strengthened their relationships because they navigated the challenges together. Others experienced significant family stress during the waiting period, which caused conflicts and disagreements, especially about treatment and social isolation due to infection control.

Some participants shared that their family members weren’t involved in their care and were unaware of the upcoming transplant.

”It was awful… this waiting period had been going on for two, three years, and it was perhaps the worst period of my life because I felt completely alone in it.” (P13).

Almost all participants frequently felt guilty for disrupting their families’ lives. The most difficult situations arose when family members disagreed about treatment and infection control.

“My parents often argued about my treatment, and it was difficult for me to hear. One of my parents would not listen to anyone and would blame my other parent if I got an infection or if my lung function did not improve after intravenous treatment. I have little contact with that parent today, as I always felt guilty.” (P5).

Participants also described guilt related to the donor. In moments of desperation, they wished for a scenario where someone might die so they could receive new lungs. One participant described this internal conflict and the psychological and existential challenges that came with being on the waiting list:

“I felt unworthy, struggling with the thought that someone else needed the lungs more than me. The guilt weighed heavily on me.” (P12).

Another participant echoed this sentiment:

“I started thinking… I had wished that some motorcyclist or someone could die so that I could get these lungs. And then I was a bit disappointed in myself in some way, that I had those thoughts.” (P7).

#### The role of communication

All but two participants described wanting a more open and transparent dialogue with healthcare professionals earlier in their interactions. They emphasized that these conversations should cover their medical condition comprehensively, including the potential for lung transplantation. This need for early dialogue was noted by those who had been transplanted many years ago and those who had undergone the procedure more recently.

Participants believed that if this dialogue had been initiated earlier in the clinical routines, they would have better understood their situation. Early conversations would have helped them grasp the seriousness of their condition and the potential benefits and risks associated with lung transplantation. Participants said this early engagement could have alleviated some of their anxieties and better prepared them for future decisions and challenges.

“It is hard to start talking about difficult things if you are never asked or have the conversation.” (P13).

However, some participants acknowledged that they must be mentally and emotionally prepared for such dialogues. They recognized that receiving and processing complex and potentially distressing information required a certain level of readiness and support. One participant shared their experience, highlighting the importance of preparation:

“For a long time, I was not ready to hear about the possibility of transplantation. It was overwhelming to think about. However, when my doctor finally sat me down and explained everything clearly, I felt more in control and less anxious about my future. I wish this conversation had happened earlier, but I also needed time to come to terms with my condition.” (P15).

Participants expressed needing more proactive communication and comprehensive information to increase their understanding of potential long-term side effects after transplantation and the risks associated with immunosuppressive medications.

“I wish I had received more detailed information about the side effects you can get as I had no idea that you could be affected by such things.” (P21).

Participants emphasized the importance of not treating guilt and the wish for another’s death as taboo, recognizing how natural it is to have such thoughts and feelings in a severe situation. They acknowledged that facing a life-threatening illness and trying to cope can lead to complex and sometimes uncomfortable thoughts. They highlighted the need to discuss these feelings openly and without judgment as a crucial aspect of emotional support during the transplant process. One participant shared:

“While no one wishes for anyone’s death, such thoughts may cross one’s mind when one is seriously ill.” (P7).

### Reborn with new lungs

#### A second chance but with new challenges

When participants woke up after the transplantation, they first felt appreciation for life and the ability to breathe freely and effortlessly. The newfound ease of taking deep breaths felt like receiving a gift or being granted a second chance.

“I took a deep breath… then I held it all the way down to my toes. And then I did it again…” (P12).

However, the time after transplantation was not without difficulties. All participants, except two, described experiencing extreme pain after the lung transplantation and not being prepared for this intensity of pain when waking up in the ICU after surgery.

“Pain like hell.” (P11).

…when I asked before [the transplantation], will it hurt? No, no, you will get enough painkillers… but that was not true.” (P16).

Participants also described struggling with pain several years after the lung transplantation. Pain made them feel weak and incapable of performing the recommended activities, such as physical training. Some also felt that healthcare professionals did not take their concerns about pain seriously.

“It is several years after the lung transplantation… I still have terrible tingling in my feet and legs and pain in my calves, especially at night. I have a numbness like a belt around my armpits over all the incisions. I have no feeling in the front of my chest, and it is painful.” (P5).

“I have problems with pain in my legs and feet and problems walking, and I have had it since transplantation five years ago.” (P21).

Many participants shared the challenges of depending on family members for daily tasks and emotional support, often resulting in feelings of guilt. This reliance underscored the emotional complexities of recovery as participants struggled to reconcile their evolving identities with the reality of needing assistance during the healing process.

“I felt bad for needing so much help; I didn’t want to burden my family, but I couldn’t manage on my own.” (P7).

Extended periods of sickness consumed much of their physical and mental energy, leaving little room for addressing deeper, more personal concerns. Participants reported primarily focusing on managing CF and its immediate impacts. As a result, once they started to recover and had more energy and time, they became more acutely aware of these neglected aspects, such as existential thoughts, leading to feelings of depression and anxiety.

“I have felt like this afterward that a lot of my, if you want to call it, mental problems—it is not that I should call it mental problems, but like depression, anxiety, and that kind—it has come after the transplant when I felt better and had more time in some ways to focus on things that I had to ignore because I did not have the time and energy to think about things like that.” (P16).

#### Adjusting to a new identity

Following a lung transplant, participants reported a mix of emotions as they transitioned from a life bound by daily inhalations and intravenous treatments to one of newfound freedom. These integrated routines had become a significant part of their lives, and their sudden removal provoked a re-evaluation of their expectations and a redefinition of their sense of self. For some, this liberation was tinged with unfamiliarity, as they had become accustomed to their patient role, and adherence to medical regimens had become second nature.

“Sometimes I do not recognize myself, like when I stand in front of the mirror. Before the transplant, I always had oxygen in my nose: I was thin and had an infusion pump with antibiotics. Now, nothing…” (P2).

Many participants noticed a significant shift in how others perceived them following their transplant. On the one hand, they experienced a sense of liberation due to their improved health status. They enjoyed the newfound freedom to engage in previously impossible activities, enjoying a better quality of life and the ability to participate more fully in social and physical activities. On the other hand, this change also came with its challenges. Participants felt subtle pressure to meet the new expectations from their improved appearance and health. Seeing the outward signs of recovery, family, friends, and colleagues often assumed they were fully capable and free of any persistent issues. This assumption sometimes led to unrealistic expectations regarding their energy levels and capabilities, causing participants to feel stressed and overwhelmed as they tried to live up to these new standards:

“CF is a serious disease like dealing with cancer. Being transplanted, you do not have the same diagnosis or the same fear of dying, but you switch one serious condition for another.” (P5).

### A new life with medications and their effects

#### Emphasizing the importance of medications

Participants acknowledged the critical role of immunosuppressive medication in preventing organ rejection. They described understanding the necessity of adhering to their medication regimen.

” I would not dare to risk rejection by messing with the medication and taking a chance because I do not want to take it.” (P5).

For those who have spent their lives managing CF and its associated treatments, the transition to immunosuppression therapy after transplantation was not markedly challenging. Most participants found it relatively easy to incorporate the immunosuppressive regimen into their pre-established medication routines.

“It has not been a huge difference… taking the pills is obvious stuff.” (P8).

Despite understanding the severe consequences of non-adherence, such as organ rejection, participants described instances of missed doses. They frequently attributed these lapses to competing priorities, including work obligations, social events, and family responsibilities.

“I was doing well during sick leave, but I became careless once I returned to work.” (P9).

#### Burden with side effects

Each day became a balance between feeling grateful for the new lungs and managing the discomforts caused by the immunosuppressive medication. The participants described struggling to manage the side effects.

“Side effects have darkened my life after the transplant, I must say. But I am finally on a medication that’s somewhat effective, even though my stomach has been very troublesome” (P3).

Another participant noted that many transplanted persons tend to downplay their health struggles because they perceive them as minor compared to the life-altering experience of undergoing a transplant:

“I have noticed, which has surprised me a little, that the side effects are greater than I thought. Many of us transplant patients feel that what is happening now is nothing compared to having undergone a transplant, so we do not talk about it. It seems to be something, but if you scratch the surface a little, this and that and the third come up.” (P9).

Participants expressed that they experienced various side effects that significantly impacted their lives. They felt that healthcare professionals did not fully understand their concerns, as the focus primarily was on the new lungs and CF.

“They did not understand me. They sent me to a psychologist and a physiotherapist because I was tired and weak. One day, I had a high fever and went to the emergency ward at the hospital. They took extra blood samples, which showed that I had lymphoma. It felt somewhat good because no one relied on me.” (P18).

Other participants described severe complications related to immunosuppressive medication. One participant shared:

“My kidneys collapsed, I am blind in one eye, and I developed skin cancer.” (P21).

## Discussion

The present study is one of few that seeks to gain a deeper understanding of the lived experiences of individuals with CF who have undergone lung transplantation, focusing on both the waiting period (pre-transplant) and the post-transplant experiences. Participants shared various physical and psychological challenges faced during both phases, including fatigue, reduced strength, depression, and anxiety, as well as feelings of dependency, isolation, and guilt.

Additionally, they experienced family stress, anxiety, and depression. Uncertainty during the waiting period heightened anxiety about their health and the transplantation process. This emotional burden underscores the critical need for psychological support as an integral part of the transplant process. These findings align with those from other studies on organ transplantation conducted by Kuntz et al. [24].

After the transplantation, participants struggled with adjusting to a new identity, managing physical pain, and coping with unexpected side effects. Many emphasized better communication and preparation for the transplantation process, particularly concerning immunosuppressive medication and post-surgery expectations. These findings align with previous research on kidney transplantation by Burns et al. [25] and lung transplantation by Seiler et al. [26], which similarly noted the profound challenges transplant recipients face.

Emotional challenges were common, with participants experiencing isolation, guilt, and increased family stress due to the need to avoid infections and rely on family for support. These findings are consistent with studies by Andersson et al. [27] and Ivarsson et al. [28], which also observed similar emotional struggles among lung recipients. Feelings of guilt stemming from the disruption of family life and the sacrifice of the donor were reported, echoing results from kidney and liver transplants by Doi et al. [29] and liver transplants by Lieber et al. [30].

A key finding in this study is that most participants found adapting to a new identity challenging. The emotional complexity of transitioning from a life dependent on intravenous treatments and oxygen to one with a changed body image reflects the difficulties in recognizing and accepting oneself after the transplant. This mirrors findings by Seiler et al. [26] and Lundmark et al. [31], who explored similarities in lung recipients with various lung diseases, including CF. Lindberg et al. [32] further demonstrate that heart transplant recipients also face challenges adjusting to post-transplant limitations and a transformed body.

Another significant issue was acute pain when waking up in the ICU after the transplantation, which they were not prepared for, emphasizing the need for more thorough preoperational education on postoperative pain management. This finding is consistent with previous research by Loxe et al. [33]. For nurses, this highlights the importance of enhancing preoperative education to better prepare patients for postoperative pain, thereby improving patient outcomes and fostering more vital patient-nurse communication.

Most participants found it relatively easy to transition to immunosuppressive medication, which they attributed to their lifelong experience managing daily treatment for CF. This contrasts with findings that reported difficulties in medication adherence among kidney recipients [34, 35]. The reduced treatment burden of post-transplant for individuals with CF may explain this difference.

Participants in this study were also concerned about unexpected side effects of immunosuppressive medication, such as skin problems, decreased kidney function, leg pain, and muscle weakness– consistent with findings from Lundmark et al. [36] on lung recipients and Smith et al. [37] on kidney recipients. Many reported experiencing ongoing pain years after surgery, with some feeling that healthcare professionals did not take their concerns seriously. This finding corroborates with several studies on chronic pain in transplant recipients [38–41].

A central finding of the study is the importance of timely, comprehensive, and transparent communication about the transplantation process. Participants highlighted the need for early and detailed discussions about the procedure regardless of how long ago their transplantation occurred. This is consistent with recent research that underscores the importance of early discussions, particularly for individuals with CF [42]. Similarly, Smith et al. [37] found that earlier introduction to transplantation improves psychological outcomes. In contrast, Mestres-Soler [43] noted that open and transparent communication and personalized care made participants feel well-informed and supported. Integrating palliative care into post-transplant treatment has the potential to enhance patients’ quality of life and well-being by addressing their physical, psychological, social, and spiritual needs [44]. For example, palliative care has a focus on pain management, emotional support for anxiety and depression, and assistance with navigating complex medication regimens, which may thereby improve overall patient satisfaction and coping strategies. This study highlights the importance of integrating a palliative approach to enhance overall well-being and effectively manage symptoms. It reinforces the idea that palliative care is not about treating recipients as if they are dying but rather about providing holistic support for physical, psychological, social, and spiritual needs at every stage of their illness. Previous studies, such as those by Pawlow et al. [45] and Nolley and Morrell [46], which advocate for palliative care as a proactive, preventive measure throughout the transplant journey, support this perspective.

Integrating a multidisciplinary team of specialists, such as psychologists, mental health professionals, and pain management experts, is important for supporting individuals as they adjust to new identities after transplantation. These professionals can address emotional challenges, identity changes, and chronic pain, contributing to the overall care of transplant recipients [46]. As West and Winnike [47] suggest, social workers can assist patients in navigating available resources and support systems, supporting a comprehensive approach to their well-being.

### Clinical implications for nurses

Nurses can play a pivotal role in providing multidisciplinary support to lung transplant recipients. They facilitate open communication between patients, families, and the healthcare team and ensure that patients are well-informed about their treatment and potential challenges [48].

Effective pain management is a critical component of post-transplant care, where nurses assess, monitor, and adjust pain relief strategies tailored to individual patient needs, improving their quality of life. This includes educating patients about pain management options and offering ongoing support during recovery. Moreover, nurses are essential in educating patients on complex immunosuppressive regimens, monitoring for side effects, and helping prevent complications, which promotes long-term transplant success [49]. Nurses also advocate for a holistic, person-centered approach that addresses emotional, psychological, and spiritual needs, helping patients cope with the stress of transplantation from preoperative anxiety to postoperative recovery [50].

Finally, nurses should support the early integration of palliative care into the transplant process, providing comprehensive support that addresses short-term and long-term challenges throughout the transplant journey [51].

### Strengths and limitations

The current study is one of the few qualitative studies exploring the experiences of individuals with CF who have undergone lung transplantation. The study had a relatively large number of participants, with only four individuals declining to participate. Another important strength is that study participants reported similar experiences and needs, regardless of age, duration on the waiting list, or years since transplantation.

No individuals with CF were on the waiting list for lung transplantation at the time of the study, which may have limited the capture of experiences from those currently awaiting transplantation. Additionally, the retrospective study design increases the risk of recall bias. The interviewer had prior interactions through providing care to some participants, which may have influenced their participation and responses, potentially introducing bias. This pre-existing relationship could have impacted participants’ openness and willingness to share candidly during the interviews.

Nevertheless, both participants transplanted recently and those transplanted more than fifteen years ago consistently expressed similar concerns about the adequacy of timely earlier information and chronic pain issues. This consistency suggests that these issues have persisted and remain relevant.

To address potential biases, the research team, which included two RNs with extensive CF care experience and two MDs specialized in respiratory diseases, engaged in reflexivity during the analysis. This process involved critically reflecting on the researchers’ roles, biases, pre-understandings, and assumptions throughout the analysis.

## Conclusion

This study underscores the significant physical and emotional challenges individuals with CF face during lung transplantation, highlighting the need for comprehensive, person-centered care. Psychological support, effective post-transplant pain management, and early palliative care may be beneficial approaches to improve the patient experience. Nurses can play a pivotal role in this process by ensuring clear communication, managing pain, educating patients on immunosuppressive regimens, and advocating for holistic care. A multidisciplinary team approach that addresses medical, psychological, and palliative needs may improve patient outcomes and satisfaction, enhancing well-being and long-term success. Further research is needed to explore these approaches and their impact on patient care and outcomes.

## Electronic supplementary material

Below is the link to the electronic supplementary material.

Supplementary Material 1

## Data Availability

The data supporting this study’s findings are available from the corresponding author upon reasonable request.

## Abbreviations

CF: Cystic Fibrosis
CFTR: Cystic Fibrosis transmembrane conductance regulator
COREQ: Consolidated criteria for reporting qualitative studies
FEV1: Forced expiratory volume in 1 second
FVC: Forced vital capacity
ICU: Intensive care unit
MD: Doctor of Medicine
RN: Registered Nurse
