# Supplementary material for: Lived experiences for individuals with cystic fibrosis who have undergone lung transplantation: a qualitative study
Source: BMC Nurs. 2025 Feb 3;24:127. doi: 10.1186/s12912-025-02774-x (PMC11792671; doi:10.1186/s12912-025-02774-x)
Supplement: Supplementary file 1 — Supplementary Material 1 [file 12912_2025_2774_MOESM1_ESM.docx]

**Research checklist**

Consolidated criteria for reporting qualitative studies (COREQ): 32-item checklist

| **Domain 1: Research team and reflexivity** | | |
| --- | --- | --- |
| Personal Characteristics |  |  |
| 1. Interviewer | US | Page |
| 2. Credentials.  What were the researcher's credentials? | US: RN, MsC  IdM: MD, PhD  TP: MD, PhD  TG: RN, PhD |  |
| 3. Occupation.  What was their occupation at the time of the study? | US: RN, PhD-student  IdM: MD, Researcher  TP: MD, Researcher  TG: Professor |  |
| 4. Gender.  Was the researcher male or female? | US: Female  IdM: Female  TP: Female  TG: Female |  |
| 5. Experience and training.  What experience or training did the researcher have? | US: Performed courses in qualitative research  IdM: Considerable experience in quantitative research  TP: Considerable experience in quantitative research  TG: Possess significant experience in conducting qualitative research across various research projects in palliative care, one of them concerned her PhD thesis (interviews with terminally ill patients in phase I trials). |  |
| Relationship with participants |  |  |
| 6. Relationship established  Was a relationship established before study commencement? | The relationship was established with some participants before the study commencement. | Page 4 |
| 7. Participant knowledge of the interviewer  What did the participants know about the researcher? | Some participants knew the US as an RN at one of the CF centers. | Page 4 |
| 8. Interviewer characteristics  What characteristics were reported about the interviewer/facilitator? | That US is interested in CF patients waiting for lung transplantation as a RN. The entire research group shares this interest. | Page 7-8 |
| **Domain 2: study design** | | |
| Theoretical framework |  |  |
| 9. Methodological orientation and Theory  What methodological orientation was stated to underpin the study? | Inductive content analysis. | Page 6 |
| Participant selection |  |  |
| 10. Sampling  How were participants selected? | Purposive sampling. | Page 4 |
| 11. Method of approach  How were participants approached? | By phone or in person. | Page 5 |
| 12. Sample size  How many participants were in the study? | 23 | Page 5 |
| 13. Non-participation  How many people refused to participate or dropped out? Reasons? | Four individuals declined participation without giving any reason. No dropout. | Page 5 |
| 14. Setting of data collection  Where was the data collected? | They were allowed to choose both the timing and location for the interviews. These interviews could be conducted in a quiet room at the hospital or via the digital Teams platform, catering to various locations and individuals, including those with compromised immune systems. | Page 5 |
| 15. Presence of non-participants  Was anyone else present besides the participants and researchers? | To the interviewer’s knowledge, no one else was present besides the participants and researchers. | Page 5 |
| 16. Description of sample  What are the important characteristics of the sample? | The participants are described in detail. | Page 4+5 |
| Data collection |  |  |
| 17. Interview guide: Were questions, prompts, and guides provided by the authors? Was it pilot tested? | Yes, semi-structured. Yes, it was pilot-tested. We conducted two pilot interviews to ensure that the interview guide was appropriate. The guide was reviewed by healthcare professionals, comprising two RNs and a psychologist with clinical experience in CF care. In addition, two people with CF who had undergone transplantation, one newly transplanted and one transplanted some years ago, provided valuable input to the interview guide. | Page 5+7 |
| 18. Repeat interviews. Were repeat interviews carried out? If yes, how many? | No. |  |
| 19. Audio/visual recording.  Did the research use audio or visual recording to collect the data? | Yes, we used an audio recorder. | Page 5 |
| 20. Field notes  Were field notes made during and/or after the interview or focus group? | No additional field notes were taken. |  |
| 21. Duration.  What was the duration of the interviews or focus group? | The interviews lasted between 12 and 46 minutes | Page 5 |
| 22. Data saturation.  Was data saturation discussed? | Yes, and we reached data saturation in the last five interviews. | Page 5 |
| 23. Transcripts returned. Were transcripts returned to participants for comment and/or correction? | No. |  |
| **Domain 3: analysis and findings** | | |
| Data analysis |  |  |
| 24. Number of data coders.  How many data coders coded the data? | The first author conducted the initial coding of the data, and the last author read eleven interviews. Discussions were held between the first and the last author. All authors met and discussed the results until a consensus was reached. | Page 6 |
| 25. Description of the coding tree. Did authors provide a description of the coding tree? | Table 3 displays the categories and subcategories identified in the analysis codes. | Page 7 |
| 26. Derivation of themes.  Were themes identified in advance or derived from the data? | The categories were not identified in advance but derived from the data. | Page 6+8 |
| 27. Software.  What software, if applicable, was used to manage the data? | Excel and Microsoft Word | Page 6 |
| 28. Participant checking.  Did participants provide feedback on the findings? | No. |  |
| Reporting |  |  |
| 29. Quotations presented.  Were participant quotations presented to illustrate the themes/findings? Was each quotation identified? | Yes. All identifiers were removed from the transcribed interviews and pseudonymized with a code to ensure that interviewees could not be identified. | Page 7 |
| 30. Data and findings are consistent.  Was there consistency between the data presented and the findings? | The authors tried to keep the findings close to the original data, and the interviewer recognized the discussions in the final results presentation. | Page 8-17 |
| 31. Were major themes clearly presented in the findings? | The major categories are described in the Results and Table 3. | Page 8-17 |
| 32. Clarity of minor themes.  Is there a description of diverse cases or a discussion of minor themes? | The subcategories are described in the text as well as in Table 3. | Page 8-17 |
